# Scaling up qualitative research to harness the capacity of lay people in invasive plant management

**DOI:** 10.1111/cobi.13929

**Published:** 2022-07-27

**Authors:** Nicholas Gill, Laurie Chisholm, Jennifer Atchison, Sonia Graham, Gina Hawkes, Lesley Head, Shaun McKiernan

**Affiliations:** ^1^ School of Geography and Sustainable Communities, Faculty of the Arts, Social Sciences and Humanities University of Wollongong Wollongong New South Wales Australia; ^2^ School of Earth, Atmospheric, and Life Sciences, Faculty of Science, Medicine and Health University of Wollongong Wollongong New South Wales Australia; ^3^ School of Geography, Earth and Atmospheric Sciences, University of Melbourne The University of Melbourne Parkville Victoria Australia

**Keywords:** environmental management, invasive plants, lay experience, meta‐ethnography, qualitative research, reframing, social science, ciencias sociales, experiencia común, gestión ambiental, investigación cualitativa, meta‐etnografía, plantas invasoras, replanteamiento, 入侵植物, 元民族志, 定性研究, 社会科学, 非专业人士的经验, 重构, 环境管理

## Abstract

Successful management of invasive plants (IPs) requires the active participation of diverse communities across land tenures. This can be challenging because communities do not always share the views of scientists and managers. They may directly disagree, have alternative views, or be unwilling to manage IPs. Reviews of IP social science identify opportunities to better understand the role of cultural processes and everyday practices to address these challenges. To scale up and leverage the insights of existing qualitative social science IP research, we used meta‐ethnography to unlock accounts and interpretations of lay perspectives. Meta‐ethnography is a form of qualitative research synthesis increasingly used beyond its origins in health and education to produce interpretive syntheses of an area of research. In the 7 phases of meta‐ethnography, we systematically identified and synthesized 19 qualitative articles pertinent to lay experience and knowledge of IPs in diverse settings. Action and meaning regarding IPs were influenced by 6 meta‐themes in personal and social life: dissonance, priorities, difference, agency, responsibility, and future orientations. Through descriptions and examples of each meta‐theme, we demonstrated how the meta‐themes are higher level structuring concepts across the qualitative research that we analyzed and we retained grounding in the in‐depth qualitative research. We characterized the meta‐themes as leverage points and tensions by which we reframed lay people in terms of capacity for reflective IP management rather than as obstacles. The meta‐ethnography synthesis shows how leverage points and tensions emerge from everyday life and can frame alternative and meaningful starting points for both research and public engagement and deliberation regarding IP management. These insights are not a panacea, but open up new space for reflective and mutual consideration of how to effectively navigate often complex IP problems and address conservation and social and livelihood issues in dynamic social and physical environments.

## Abstract 2

Aprovechamiento de la Capacidad de la Gente Común en el Manejo de Plantas Invasoras

## Resumen

El manejo exitoso de las plantas invasoras requiere de la participación de las diferentes comunidades participantes en la tenencia de tierras. Esto puede ser un reto porque las comunidades no siempre comparten las opiniones de los científicos y los gestores. Puede que las comunidades estén en desacuerdo directo con estas opiniones, tengan opiniones alternativas o no estén dispuestas a manejar las plantas invasoras. Las revisiones a las ciencias sociales de las plantas invasoras identifican oportunidades para entender de mejor manera el papel de los procesos culturales y las prácticas diarias para abordar estos retos. Usamos la meta‐etnografía para revelar explicaciones e interpretaciones de las perspectivas comunes para aumentar y potenciar el conocimiento cualitativo de las investigaciones en ciencias sociales de las plantas invasoras. La meta‐etnografía es una forma de síntesis de la investigación cualitativa que se usa cada vez más fuera de sus orígenes en la salud y educación para producir síntesis interpretativas de un área de investigación. En las siete fases de la meta‐etnografía identificamos y sintetizamos sistemáticamente 19 artículos cualitativos correspondientes a la experiencia y el conocimiento no profesional de las plantas invasoras en diferentes escenarios. Las acciones y significados con respecto a las plantas invasoras estuvieron influenciadas por seis meta‐temas en la vida personal y social: disonancia, prioridades, diferencia, agencia, responsabilidad y orientaciones a futuro. Por medio de descripciones y ejemplos de cada meta‐tema, demostramos cómo estos son conceptos estructuradores de nivel más alto a lo largo de la investigación cualitativa que analizamos y mantuvimos basados en la investigación cualitativa a profundidad. Caracterizamos los meta‐temas como puntos de potenciación y tensiones por la cual replanteamos a la gente común en términos de capacidad de reflexión en el manejo de plantas invasoras en lugar de considerarlas obstáculos. La síntesis de la meta‐etnografía muestra cómo los puntos de potenciación y las tensiones emergen de la vida cotidiana y pueden plantear puntos de partida alternativos y significativos para la investigación, la participación pública y la deliberación en el manejo de plantas invasoras. Esta información no es la panacea, pero puede abrir un espacio nuevo para la consideración mutua y reflexiva de cómo navegar efectivamente los problemas complejos de las plantas invasoras y cómo abordar los temas de conservación social y de sustento en los ambientes dinámicos sociales y físicos.

## 【摘要】

入侵植物的成功管理需要不同土地制度的社区的积极参与。这可能是一个挑战, 因为社区居民并不总是认同科学家和管理者的观点。他们可能会直接反对、持不同观点, 或不愿意管理入侵植物。对入侵植物的社会科学综述有助于更好地了解文化过程和日常实践的作用, 以应对这些挑战。我们使用元民族志来展示非专业人士观点的描述和理解。元民族志是一种定性研究综述的形式, 起源于卫生和教育领域, 已被越来越多地被用于对一个研究领域进行诠释性的综述。在元民族志的 7 个阶段中, 我们系统地确定并概括了 19 篇与不同环境中非专业人士对入侵植物的经验和知识有关的定性文章。我们发现, 关于入侵植物的行动和意义受到个人和社会生活中 6 个元主题的影响: 不协调性、优先性、差异、机构、责任和未来方向。通过对每个元主题的描述和举例, 我们展示了元主题如何在我们分析的定性研究中形成更高层次的结构性概念, 并且我们在深入的定性研究中保留了基础。我们将元主题描述为杠杆点和张力, 在此基础上, 我们将非专业人士重新定义为思考入侵植物管理的能力, 而非障碍。元民族志的整合显示了杠杆点和张力如何出现在日常生活中, 且可以为有关入侵植物管理的研究和公众参与及讨论提供有意义的替代性起点。这些见解不能解决所有问题, 但可以为反思和交互性考虑如何有效管理复杂的入侵植物问题, 以及在动态的社会和物理环境中解决保护和社会及生计问题开辟了新的空间。**【翻译: 胡怡思; 审校: 聂永刚】**

## INTRODUCTION

Invasive plants (IPs) present a major environmental threat, generate significant and growing economic costs (Bradshaw et al., 2021; Diagne et al., 2021), and are a key defining (Kueffer, 2017) and expanding (Seebens et al., 2017) feature of global environmental change. Despite agreement among researchers and other experts that IPs are problematic, disagreements on the nature of the problem and how best to deal with it are rife (Ricciardi et al., 2017; Sagoff, 2020). In an evolving set of debates, researchers have, for example, disagreed over the categorization and impacts of invasive species, alleged and refuted so‐called invasive species denialism, skirmished over whether biological invasions constitute a distinct field of knowledge, debated what counts as natural processes of species distribution, and argued over focusing on impacts relative to origins (Fall, 2021).

These disagreements are not just among researchers; the views of lay people often diverge from the views of IP policy makers, practitioners, and scientists (Courchamp et al., 2017; Kapitza et al., 2019). Moreover, effective governance and management (Appendix S1) of IPs requires participation of lay people in a wide range of circumstances and spaces (Graham et al., 2019; Head et al., 2015; Howard, 2019), many of which are practically and politically difficult for the state to access (Barker, 2010; Higgins et al., 2016). Lay participation is especially required where devolved responsibility for biosecurity is integral to policy, legislation, and institutions (Barker, 2010; Rawluk et al., 2021). Effective participation is unlikely without recognition of the significance of perceptions and understandings of IPs (Shackleton, Richardson, et al., 2019) and meaningful involvement in goal setting, planning, and decision‐making (Shackleton, Adriaens, et al., 2019). This need has been recognized by physical (Courchamp et al., 2017) and social scientists (Estévez et al., 2015; Head, 2017; Kapitza et al., 2019; Shackleton, Richardson, et al., 2019). In their review of conflicts over invasive species, Estevez et al. (2015) note that despite the framework they propose for deliberation, real‐world contexts in which “values, perceptions, and institutional issues” coalesce for people are critical. Similarly, Head (2017) points to the need to locate IPs in the everyday lives, work, concerns, and values of lay people and to improve “understanding of differences and commonalities in meanings, norms and resultant practices.” These arguments indicate the need for better understanding of the cultural processes and practices (Appendix S1) in IP social science that have proven difficult to integrate into reviews to date (Howard, 2019; Kapitza et al., 2019; Shackleton, Richardson, et al., 2019). They further indicate the role for such understanding in participatory processes to address the much critiqued but persistent “deficit model” of lay people (Cook & Melo Zurita, 2019; Shackleton, Richardson, et al., 2019). Innovative approaches suitable for these research needs are required to build on and further develop insights from these reviews and IP social science more generally (Shackleton, Richardson, et al., 2019).

Qualitative social science is well placed to examine how values, meanings, experiences, and practices coalesce in the activities and thinking of everyday life (Maller, 2021) (Appendix S1). A small but growing body of qualitative social research on IPs provides in‐depth analyses of the experiences of lay people (see METHODS for our definition of the term *lay*) regarding IPs. Qualitative researchers ask what IPs mean to people, why IPs mean what they do, and why people behave as they do in relation to IPs. The value of qualitative research derives from the methodologies afforded by its focus on small samples or in‐depth case studies and its ability to highlight the social and cultural significance of complexity, context, and contingency.

To scale up qualitative IP research and to address the opportunities for further insight into the social dimensions of IP, we conducted a meta‐ethnography in which we applied a novel (Shackleton, Richardson, et al., 2019) methodology to develop insights into the social dimensions of IP. Meta‐ethnography is a systematic qualitative research synthesis method used predominantly in education and health sciences (France et al., 2019). It is one of a range of qualitative research synthesis methods (Booth, 2016; Drisko, 2020; Noyes et al., 2022). Meta‐ethnography aims to retain and leverage the depth and richness of qualitative research across multiple publications to enhance the analytical reach of qualitative research. By aiming for interpretive synthesis (Noblit & Hare, 1988), the method may improve the role of qualitative social science in environmental research and management when “subjective experiences,” “heterogeneity,” and “context” cannot be readily addressed by systematic reviews (Macura et al., 2019). Although meta‐ethnography shares some characteristics with systematic reviews and other approaches to qualitative synthesis, meta‐ethnography is distinguished by its interpretative aims, its purposive, rather than comprehensive, sampling, and its idealist rather than realist epistemology (Drisko, 2020; Macura et al., 2019). It is “configurative” or “interpretative” rather than “aggregative,” meaning meta‐ethnographies generally aim to develop new theory or concepts to generate better understanding or interpretation of a phenomenon rather than working in an area or topic where conceptualization itself is more developed or asking, for example, what works (Drisko, 2020; Gough et al., 2012).

Thus, overall, by a systematic synthesis we sought to complement and build on insights from previous social science IP reviews and to enhance the role of qualitative research. In a general sense, we used the meta‐ethnography methodology to ask whether social categories and variables, such as those used in previous reviews, can be understood as instances of yet broader or higher order thematics of social meaning and action. More specifically, we aimed to improve interpretation and understanding of the nature and significance of IP knowledge, meanings, and experiences in everyday life among lay people, such as landowners, citizens, Indigenous people, and communities, and to consider how such insights can improve lay participation in IP governance and management (Table 1).

**TABLE 1:** Aims of the meta‐ethnography of invasive plants and lay knowledge and experience and steps and criteria for selecting studies for inclusion

Aims	Conduct qualitative synthesis of qualitative invasive plant (IP) research to improve understanding of and thereby engagement with the knowledge and experience of landowners, citizens, Indigenous people, and communities
Systematic search and initial screening	Qualitative social science research on IP; focus on lay people; search terms as per Appendix S2; 107 articles identified for further screening
Second screening	Focus on IP, lay practices, knowledge, experiences, and meanings; English language articles in a peer‐reviewed journals; qualitative methodology; 34 articles remaining
Third screening	Methodology not assessable as clearly or substantively qualitative; repetitious studies or studies based on parts of the same empirical study; did not align sufficiently with study goal; contained first author repeats; 19 articles remaining

## METHODS

Much qualitative research on IP management and governance concerns the experiences and actions of diverse publics and communities. We focused on research with those we refer to as *lay people* and this shaped our methods. We used this term to encompass the wide diversity of people and communities in the peer‐reviewed articles we analyzed and to distinguish them from scientists, policy makers, and weed management practitioners who work in agencies and organizations. The term does present some risks of being perceived to embody an expert‐deficit model of lay knowledge and to devalue local or Indigenous knowledge and experience relative to scientific, technical, and institutional knowledge and its weed management prescriptions. In contrast, our use of the term derives from a research‐based understanding that “lay publics actively create forms of understanding and knowledge as they negotiate the conditions of everyday life” (Petts & Brooks, 2006).

**FIGURE 1:**
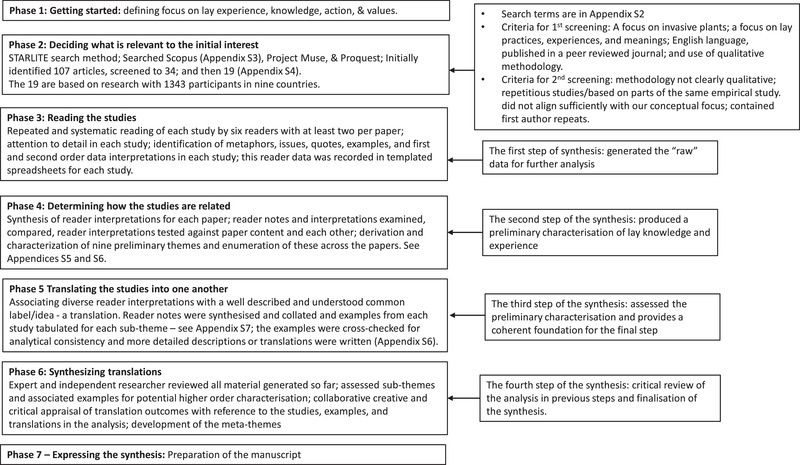
Summary of actions and purpose at each phase of the meta‐ethnography of invasive plants and lay experience and knowledge

Meta‐ethnography has 7 phases (Noblit & Hare, 1988) that derive first‐ (study participants’ data) and second‐order (authors’ interpretations) interpretations of the texts. We followed these phases as refined by France et al. (2019) (Table 2), adapting them to the specifics of our study (France et al., 2019) (Figure 1).

**TABLE 2:** The 7 phases of Noblit and Hare's (1988) meta‐ethnography approach

Phase	Description
1: Getting started	“This involves identifying an intellectual interest that qualitative research might inform.”^a^
2: Deciding what is relevant to the initial interest	The studies included need not be exhaustive. “Deciding what studies or accounts are relevant involves knowing who the audience for the synthesis is, what is credible and interesting to them, what accounts are available to address the audiences’ interests, and what your interests are in the effort.”^a^
3: Reading the studies	“The repeated reading of the accounts and the noting of interpretative metaphors. Meta‐ethnography is the synthesis of texts; this requires extensive attention to the details in the accounts, and what they tell you about your substantive concerns.”^a^
4: Determining how the studies are related	Studies are “put together” by creating “a list of the key metaphors, phrases, ideas, and/or concepts (and their relations) used in each account and to juxtapose them.”^a^
5: Translating the studies into one another	“The metaphors and/or concepts in each account and their interactions are compared or ‘translated’ within and across accounts while retaining the structure of relationships between central metaphors/concepts within accounts.”^b^ “Translations are a key synthesis step as they “protect the particular, respect holism, and enable comparison.”^a^
6: Synthesizing translations	“Parts” derived so far are made into something more than they “imply.” “[T]he various translations can be compared with one another to determine if there are types of translations or if some metaphors and/or concepts are able to encompass those of other accounts…a second level of synthesis is possible, analyzing types of competing interpretations and translating them into each other.”^a^
7: Expressing the synthesis	“Tailoring the communication of the synthesis to the intended audience's culture and language so that it is intelligible and meaningful to them.”^b^

^a^ Quotes from Noblit and Hare (1988, pp. 27–29).

^b^ Quotes and table layout from France et al. (2014, p. 3).

Searching qualitative literature for reviews and syntheses is challenging, and a systematic and transparent approach that is fit for purpose is therefore important (Booth, 2016; Drisko, 2020). Accordingly, we used the STARLITE method for our initial literature search (Booth, 2006, 2016). Our searches were purposive, seeking all available studies relevant to the focus of the meta‐ethnography (i.e., qualitative social science research on IP relating to the experiences of lay people) (search terms are in Appendix S2; see also Table 1). With both breadth and specificity, our search terms aimed to overcome the difficulties of defining search terms for qualitative syntheses, such as identifying studies by research design (Booth, 2016; Drisko, 2020; Noyes et al., 2022). As supplementary strategies (Booth, 2016), we also used snowball sampling from key review articles and included 2 searches on invasive animals for leads. For choosing databases, we were mindful that all databases have pros and cons (Gusenbauer & Haddaway, 2020; Shackleton, Adriaens, et al., 2019). Among suitable primary multidisciplinary databases, Web of Science and Scopus have similar coverage but key differences (Appendix S3). Cognizant of these differences and of our social science focus, we used Scopus as the primary search engine. We replicated the searches in ProQuest, a multidisciplinary search engine, and Project Muse, a humanities and social science database. This search generated 107 studies likely to fit the criteria of encompassing qualitative research and lay people. As recommended for meta‐ethnography (Booth, 2016), our strategy then became more purposive. We screened the initial 107 articles and deemed 34 as possible inclusions according to the criteria in Table 1 for the second screening.

Most meta‐ethnographies limit themselves to 20 articles (Britten & Pope, 2011) or less (Booth, 2016) due to the relatively high level of resources needed to adequately synthesize empirical studies with meta‐ethnography (Booth, 2016; Noyes et al., 2022). For these reasons, we next assessed the 34 articles to reduce the number. Some of us conducted closer reading of the 34 articles and made deletions based on 1 or more of the third screening criteria as in Table 1. Nineteen articles with over 1343 participants from 9 countries remained for inclusion in the meta‐ethnography (Appendix S4). The evident bias toward affluent, English‐speaking countries is noted in other reviews (e.g., Howard, 2019); it remains a challenge.

The reading phase (phase 3) was carried out independently by 6 of us, and each paper was read by at least 2 of us. We each used an identical excel spreadsheet to record information relating to geographical area; types of participants; demographic details of participants; methods used; key concepts, metaphors, and themes; stated aim of the article; recommendations or key findings and arguments; key first‐order quotes (from study participants); key second‐order quotes and findings (from article authors); and notes on other factors deemed important.

Articles were assigned to authors to ensure there was variety in publication year and that authors would not review their own articles. All 19 articles were read by at least 1 of us. Another of us read 12 of the 19, and 4 of us read a different selection across the 19. This allowed for a range of interpretations and a consistency check. One of us collated the spreadsheet data, undertook an initial assessment, and identified generalized concepts and themes in consultation with another author.

In phase 4, we consolidated the identified concepts, issues, and key arguments to provide greater clarity to the developing analysis. We undertook an extensive period of clarifying, and collectively and iteratively appraising and testing the 6 readers’ interpretations of concepts, themes, and key arguments until we reached consensus. We coded the readers’ analyses into 9 draft analytical themes (i.e., subthemes). For each paper, we generated a synthesized spreadsheet of reader notes by subthemes (Appendix S5 shows an example). We could see then how the articles related to the subthemes and how the articles were related to each other based on the distribution of subthemes (Appendix S6). Unlike other meta‐ethnographies (Britten & Pope, 2011), the articles did not fall into clear groupings of related articles. This showed that the subtheme of lay knowledges, values, and concerns was a key connecting subtheme; it was present in all but 1 paper. Other key connecting subthemes were plant–human relationships, agency and performance, and tensions and differences.

Phase 5 (translation) involved comparing the identified concepts or issues across articles. Translation is used to determine whether concepts that have different labels are nonetheless describing the same idea (Britten & Pope, 2011). This phase thus relates and compares the articles directly with each other, rather than relating each paper individually to the initial concepts, themes, and key arguments. The translation step is key to retaining the significance of the contexts of qualitative research because it is comparative rather than aggregative and thus ensures the analysis remains grounded in the studies (Noblit & Hare, 1999). To do this, 2 of us created spreadsheets for each subtheme and populated these with readers’ related concepts and issues and with examples from each paper (example in Appendix S7). With reference to the examples, we evaluated each concept or issue across the articles and considered the extent to which the subtheme was a valid representation of that concept or issue across the articles. This process systematically develops descriptions of the subthemes that are translations of the examples from the articles and of the reader‐identified concepts and issues. We described each subtheme in more detail (summary of descriptions and associated issues in Appendix S6) than in phase 4. Through translation, we determined that our subthemes were valid labels and representations of the examples (Appendix S5) and we retained them. This was a key foundation for the next phase.

In the synthesis phase, an author who was not part of previous phases critically synthesized our reader data, subthemes, and translations with fresh eyes and expert knowledge. With our goal of determining whether lay experience and knowledge of IP can be understood in terms of higher order thematics of social meaning and action in mind, the researcher examined our analyses. This author's questions drove a reevaluation of the analyses and subthemes, including how they connected to the particulars of the studies. In this process, we identified potential higher order concepts. For example, a discussion on care included the idea of priorities, led us to reconsider and reframe responsibility, and caused us to think about care for IP as coexisting dissonantly with knowledge of their impact. Priorities, responsibility, and dissonance were all developed as ideas through this process and are 3 of our meta‐themes. We used the author's analysis and this process to identify and describe 10 initial higher level concepts. Between this author and 2 others, we interrogated our use and characterization of the subthemes and how they related to these 10 higher level concepts. We thereby refined the 10 initial higher order concepts to the 6 meta‐themes.

As Noblit (2019) argues, meta‐themes arising from this synthesis process should result in a “storyline” that provides a fresh interpretation of the phenomena in the chosen readings. The preparation of this paper thus represents stage 7 of the meta‐ethnography in which we provide a storyline, represented by the meta‐themes, suitable for an audience unfamiliar with qualitative research.

## RESULTS

Our meta‐ethnography of 19 qualitative IP studies (Appendix S4) generated 6 meta‐themes (Table 3) that demonstrated coherence without losing the nuance of the individual studies. The meta‐themes represented higher level structuring concepts in qualitative IP research, distinguishing our analysis from previous reviews of social science IP research that identify gaps relating to cultural issues and processes. To preserve the rich, contextualized, and nuanced data and research at the heart of IP qualitative research, we considered the meta‐themes and for each provided examples that are diverse but comparative.

**TABLE 3:** The 6 meta‐themes derived from the meta‐ethnography analysis of invasive plants and lay experience and knowledge

Theme name	Theme characterization	Articles represented*
Dissonance	Lack of agreement, consistency, or harmony; tensions and inconsistencies that people or entities may hold, demonstrate, or experience; dissonance is pervasive and exists in individuals and entities and between them	9, 2, 3, 4, 7, 13, 14, 16, 17, 18
Priorities	Diverse, competing, and key to power dynamics in and between state, expert, and lay people; deeply embedded in existing value systems, emotional responses, and personal preferences and beliefs	1, 3, 5, 7, 9, 14, 16, 17
Difference	Weeds and other plants manifest, expose, and manufacture differences in relationships between people and between people and plants by making them materially apparent	1, 2, 8, 9, 10, 11, 15, 16
Agency	Held and exercised by both humans and plants; considers consequences of acknowledging a multi‐agentic world and what possibilities might open up by decentering human agency for plant agency and state agency for lay agency	1, 4, 6, 8, 10, 13, 17
Responsibility	Felt, distributed, and allocated in ways that match diverse understandings of law, property, nature, and plant mobility and that challenge conventional boundaries and spaces of private, public, individual, and collective responsibilities	8, 9, 12, 19
Future orientations	People forecast outcomes of their weed work, including whether they see it as meaningful, futile, as addressing uncertain environmental futures, as part of a common good, or as reflecting their own personal values and goals	3, 10, 11, 16, 19

* See Appendix S4 for a numbered list of articles in the analyses.

### Dissonance

Dissonance was deeply embedded in IP management and was the most common meta‐theme. It encompassed all the subthemes and was evident across 10 of the articles. *Dissonance* referred to a lack of consistency or harmony and to tensions and inconsistencies people or organizations held, demonstrated, or experienced. Dissonance included how people experienced IP management and navigated tensions between knowledge that a plant is invasive, that it may be officially designated as problematic, and their own experiences or uses of that plant. Such experiences or uses acknowledged the problems associated with IPs and recognized limits in human capacity to manage it. Acknowledging a plant as problematic sometimes coexisted with valuing it for certain purposes and thus deciding to retain it. For example, in suburban Australia, householders retained and “tolerated” mature camphor laurel trees (*Cinnamomum camphora*) in their gardens, even though they are a highly invasive plant, because they are difficult to remove and provide shade and opportunities for swings and tree houses (Head & Muir, 2004).

Dissonance also existed between individuals and entities. It was present in the way the state and its agents viewed themselves as enacting community‐based action and achieving success and in how communities perceived state‐centered governance as being driven by measures of success they did not equate with success, eroding commitment to IP management programs. For example, in addition to material IP control outcomes, landholders in rural Australia valued intangible outcomes of community‐based IP management programs, such as sharing knowledge, developing relationships, and inculcating shared norms around IPs (Graham & Rogers, 2017). In northern Australia, Indigenous rangers were frustrated that the natural resource management programs their work was part of drove them to undertake control work that they knew was likely to be ineffective at site and landscape scales. At the same time, Indigenous rangers were often constrained in their ability to undertake IP control at sites of cultural or social significance (Bach et al., 2019). In both cases, lay perspectives emphasized community capacity building and aspirations that derive longevity and effectiveness by targeting social goals, whereas agencies emphasized control based on plant origins and evaluated programs on weed density reduction and numbers of landholders involved.

### Priorities

IPs were tied up in a myriad of competing priorities for lay people and the state. Priorities were diverse, competing, and key to the power dynamics at play within and between state, experts, and lay people and were often deeply embedded in existing value systems, emotional responses, the structure of land ownership, and personal preferences and beliefs. Priorities were not necessarily dispassionate and were deeply embedded in the values of care for family, land, sites, and landscape tied to notions of identity, aesthetics, and the meaning of places (Trigger & Mulcock, 2005). Priorities as a theme considered which IPs were important to different peoples and agencies, who got to set priorities, and whose priorities had sway. It entailed the different priorities people had, how they perceived the priorities of others, and how this affected them. For example, in 1 study, despite regulation and exhortation regarding weed management by government agencies, lay people perceived a lack of political will to meaningfully prioritize and fund weed management, including on government land, and this affected how much they themselves prioritized it (Davis & Carter, 2014).

Priorities were diverse, socially and environmentally construed, changing, and changeable. Changing landownership for example, such as trends to amenity ownership of rural land in the United States and Australia, reshaped priorities and the relations between people and nature that underlaid the priorities (Cooke & Lane, 2018; Klepeis et al., 2009). Further, rather than existing a priori, priorities sometimes emerged from collaborative and deliberative community processes and institutions, such as those of community‐based IP management (Graham & Rogers, 2017). Priorities represented the seemingly obvious and showed up less obvious gaps between perceptions and practices. For example, when priorities were the outcome of hard‐won experience as to the very real difficulties of weed management among concerned and engaged landholders, there was greater acceptance of IPs and a considered reallocation of their resources (Ma et al., 2018).

### Difference

IPs manifested, exposed, and created differences in relationships between people and between people and plants by making them materially apparent. *Difference* contrasted with *dissonance* in that difference encompassed stronger or clearer distinctions regarding views and treatment of plants and between various groups of people. Although disagreement or conflict sometimes manifested through differences, we did not perceive the often‐internal tensions that are key to dissonance. Differences in how people saw the world materialized through IPs at various scales. At the microgeographic scale, different plants, including invasive and non‐native species and other plants officially considered weeds, were valued and accepted in some areas of suburban gardens and unacceptable in others, such as in New Zealand (Doody et al., 2014). At the landscape scale, different lay people, experts, and the state had their differences exposed. For example, there were differences between Australian rural landholders who recognized a collective interest in IP management and those who did not (Davis & Carter, 2014). Finally, different views toward belonging materialized through IPs. For example, plant–human relationships took a moral tone, such that Australian and Norwegian householders perceived their choice to leave IPs be as “generous” (Head & Muir, 2004; Qvenild et al., 2014). In such instances, these impulses were sufficient to counter official narratives that the plants in question did not belong or official narratives were seen as mean‐spirited or “puritanical” (Qvenild et al., 2014) relative to the perceived and felt openness embodied in acceptance by lay people.

### Agency

Agency drew attention to a multi‐agentic world in which human agency was decentered, where both loss of control and learning to coexist with IPs occurred and where the agency of lay people in forming their own rationalities was evident. Agency as a theme thus pointed to the inversion or dissolution of hierarchies in IP governance and management (i.e., between humans and nature, experts and nonexperts, and the governed and those who govern). It also pointed to the erosion of relative certainties of IP management in favor of a more emergent and co‐produced conceptions of IPs themselves and of human entanglements with them. For example, IPs and people's connections with them emerged from the behavior and context of a plant at varying scales. In Australian and New Zealand yards, plant behavior shaped lay understandings of plant belonging (Doody et al., 2014; Head & Muir, 2004) (e.g., whether a plant spread or not). In heterogeneous rural landscapes, plant mobility, in concert with program design, social institutions and relations, and the observed outcomes of IP management labor, collectively shaped Australian landholder understanding of the boundaries of their capacity to control IPs, leading to actions ranging from removal to tolerance of plants (Cooke & Lane, 2018). The thinking, practices, and experiences that emerged with concrete and actualized potentials of plants and the recognition of human limits in turn conditioned how lay people in several countries perceived the state, experts, and institutions of IP governance, influencing how they assessed official prescriptions for IP management (Brenner, 2011; Cooke & Lane, 2018; Ernwein & Fall, 2015).

### Responsibility

Responsibility at its simplest was doing something about IPs that meets legal or moral expectations (Setten, 2016). However, among lay people responsibility was felt, distributed, and allocated to match diverse understandings of law, property, nature, landscape history, and plant mobility and distribution. In engaging with the idea of responsibility, how it was generated, and where it lay, people actively and contextually considered relationships between themselves and others, between citizens and the state, and between humans and plants. Lay people considered how they were or were not recognized or rewarded for doing weed management in Australia (Graham & Rogers, 2017). They also perceived the complexity of responsibility when IPs are mobile and move across land boundaries, especially in heterogeneous landscapes. For example, U.S. landholders sometimes had expectations that their neighbors should control IPs but acknowledged that IP mobility meant that coordinated action was also needed and blame was not an adequate response (Fischer & Charnley, 2012). The attribution of blame sat uneasily amidst recognition that IPs are mobile and often difficult to control. However lay people also apportioned blame where governments or other landholders were seen as not taking their fair share of responsibility in countries such as the United States and Australia (Cooke & Lane, 2018; Ma et al., 2018). They also did so where governments were perceived as creating IP governance frameworks that were incommensurate with the nature of the problem and thus were not perceived by lay people as acting responsibly. Thus, the extent to which others, including governments, took responsibility was important in facilitating or discouraging weed management (Jevon & Shackleton, 2015) and shaping responses to the state (Brenner, 2011; Davis & Carter, 2014). Responsibility also encompassed how and why people did or did not take responsibility for weed management outside of or beyond legal responsibilities or where it affected their land (Jevon & Shackleton, 2015; Shrestha et al., 2019). In Nepal, in the absence of government action, lay people attempted to control emerging IP problems on commonly managed land, such as forests (Shrestha et al., 2019), whereas in a comparable South African case, steps were not taken despite similar impacts on forest products (Jevon & Shackleton, 2015). These examples pointed to different circumstance and pathways by which responsibility was felt or not and then acted on or not and suggested the role of local sociocultural or institutional factors in shaping this. Finally, as in Scotland, responsibility was connected to agency because lay people perceived that responsibility may vary in light of their assessment of how controllable a weed is or the manner in which it was introduced to an area (Selge et al., 2011). For example, they sometimes distinguished between introductions by humans and introductions by “natural spread” (Selge et al., 2011) and made moral judgements regarding responsibility where introduction by humans occurred.

### Future orientations

People forecasted the outcomes of their IP work, including whether they saw it as meaningful, futile, part of uncertain environmental futures, a common good, or reflecting their own personal values and goals. Future orientations in IP management were shaped by how people looked at creating future environments that embodied changed relationships with land and nature. In postcolonial nations, for example, this included settlers looking to make and be part of restitution for a harmful past. They looked forward to creating a personal legacy and less unsettled, more at‐home futures in which ecological hybridity is accepted as not necessarily and ideally desirable but certainly inevitable and as consistent with a future in which such nations, such as Australia, are more reconciled with their past (Trigger & Mulcock, 2005). In orientations to the future people melded interpretations of the past with their sense of control, or lack thereof, over IPs and a view that it was too late to worry about some plants. Thus, people, such as in New Zealand and Norway, perceived that IPs will be part of future environments that always require care, tolerance, or removal depending on context (Doody et al., 2014; Qvenild et al., 2014). Broadly, future orientation encompassed fears and anxieties about environmental futures and thinking about future consequences at the personal and familial levels, as well as at larger social and global scales. What to do about and how to think about IPs were integral parts of anxieties and thoughts about the future.

## DISCUSSION

Our analysis showed that by inviting citizen engagement in invasive species management and, in some jurisdictions, invoking shared responsibility as a pillar of governance, state actors actively engage lay actors who will not slavishly subscribe to science or state‐sanctioned formulations of IP risk and management. Nonetheless, the active participation of lay people is needed across many spheres of IP management: managing and controlling IPs on private land, volunteering on public lands, undertaking surveillance, practicing weed hygiene, accepting plant removal from public spaces, and choosing plants for gardens (Graham et al., 2019). Our results across 9 countries and diverse species, land uses, and settings showed that lay actors will be active, reflective, and questioning participants in public and private IP management and governance. They will exercise capacity to weigh risks, distinguish between feasible and infeasible control, and propose different approaches and means of achieving biosecurity goals.

The meta‐themes provided a means to interpret these responses and to understand them in relation to broader spheres of social concern and action. Looking ahead to potential transitions in IP governance and management, the meta‐themes are a bridge. They can help researchers, policy makers, and government authorities move from seeing lay people as essentially problematic and as obstacles, to drawing on them as a resource for more substantive engagement on terms that are meaningful to them.

The meta‐themes intersected to different degrees and should be considered as bundled rather than distinct. Nonetheless, they can be categorized in 2 groups: leverage points (agency, responsibility, and future orientations) and tensions (dissonance, difference, and priorities). With leverage points there is relative potential for productive engagement with lay people, whereas with tensions there is potential for obstructions to productive pathways, indicating the need for deliberation and further research to determine potentially constructive roles.

Leverage points are a metaphor referring to where interventions for change can be made and where diverse “relational values” that people already hold can be harnessed (Chan et al., 2020). In leverage‐point schemas, emergent human values, goals, knowledge, and worldviews have the most potential to effect productive change or trajectories (Abson et al., 2017; Chan et al., 2020; Meadows, 1997). Our results showed that in IP management by lay people, these social elements are emergent and, although their form may be recognizable, their background and substance are contextual. Agency, for example, was a leverage point because this was where lay knowledge developed in context and where people learned from each other and through their experience of plants and the landscapes they lived and worked in. Responsibility was a leverage point because it provided a way to engage with the terms on which responsibility formed and through which responsibility was apportioned. Future orientations were a leverage point as the locus of hope and ideals for futures that were to be created, including by lay people. Future orientations, in conjunction with awareness of the challenges of extant IPs, suggested that people were potential allies in preventing future IP problems. Although leverage points may ostensibly be familiar and appear transparent (e.g., different views on an IP), their substance—how views about IPs were formed, who should take responsibility, and how they should be managed in future—is not necessarily intuitive (Meadows, 1997).

The leverage points we have identified constituted unspoken ways of knowing, experiencing, and acting on IPs. They arose from intertwined biographies of people, place, land, and plants. The value of qualitative methodologies that engage with this heterogeneity and social complexity has been demonstrated, for example, by sustainability researchers using such methodologies to delineate change points—“moments in everyday routines where different courses of action can be taken” (Watson et al., 2020). These insights have been valuable for U.K. researchers and policy makers to engage in structured dialogue to reframe policy problems and courses of action (Watson et al., 2020).

Where meta‐themes embody tensions, there is a need for careful pathways to deliberation, problem definition, and investment in governance institutions, processes, and interventions. Tensions were not wholly negative or without relevance to enhanced governance and management. Dissonance and difference, for example, encompassed awareness of trade‐offs and desires for realistic and targeted IP management. Priorities brought values, emotions, and identity into deliberations about what plants to focus on and how and where to manage them. In such instances, problem definition itself is likely to be a key focus of any pathway to intervention; invasive species often represent wicked problems in which technical problem framing and solutions alone can be inadequate, elusive, and potentially counterproductive (Woodford et al., 2016). For example, in Australia the process used to list Weeds of National Significance has been described as “best practice and scientifically sound” (Wild Matters, 2020), but exclusion of nonscientific and nongovernment stakeholders from the process has led to concerns about bias and mistrust (Wild Matters, 2020). Despite their potentially important role, it is clear that pathways framed by tensions are likely to be more challenging than those framed by levers. Both leverage points and tensions can be indirect. That is, they can be based in realms of social life not directly related to IPs or even to natural resource matters at all. Identifying such leverage points and tensions and their relevance is valuably done via the open methodologies and methods of qualitative research to produce distinctive, significant, and consequential knowledge (Spotswood, 2016).

The 6 meta‐themes we identify through our meta‐ethnography constitute a novel conceptualization of tensions and leverage points in IP management. They speak to alternative, consistent, and widespread lay rationalities of IP assessment and management. Our results showed that these alternative rationalities are not simply outcomes of deficits, such as ignorance or lack of will to action. Nor do they necessarily represent resistance to the expertise and authority of the state and science, or even denialism. Rather, they represent capacity—inquiry, reflexivity, experimentation, observation, and engagement—to navigate this problem in the context of uncertain environmental futures and to strengthen the public good values that IP management embodies. Importantly, they illustrate that, like scientists and agency staff, lay people have the capacity to perceive the complexity of IP management and to respond constructively. We found that there remain opportunities to provide resources for and design IP governance and management to harness this capacity to a greater extent.

Disagreements about how to frame the problems associated with IPs and how to manage them continue. Even as they depend on participation by lay people, IP managers will continue to be challenged by contrary views, variable management foci and effort, and apparent and actual apathy. There are no easy answers and no risk‐free governance and management pathways. In some cases, eradication may be possible and desirable. However, commonly and inevitably, management goals will focus on some sort of containment or reduction of adverse consequences, tempered by the benefits that IPs bring in the case of some plants and in some contexts. However, goals and consequences are defined both scientifically and socially. They may be agreed on and shared between and among researchers, managers, communities, and lay people. For example, as we found, lay people may desire to retain, or be tolerant of, IPs in certain spaces and for reasons that make sense to them but not to managers or institutions. In these circumstances one needs to know more than the values and beliefs regarding IPs—behavior is shaped by more than such parameters (Maller & Strengers, 2015). One also needs to know how IPs fit within the broader social, cultural, and spatial contexts of lay people's lives, families, and communities; how IPs sit amid their concerns for each other and for the future; how lay people juggle ideas of how to live responsibly with sometimes competing and more immediate demands; and how their views and experiences of IPs have been shaped. In principle, decision‐making according to context is already integral to IP governance and management; risk assessments in specific settings are part of IP governance and management (Lodge et al., 2016). The meta‐themes provide scaffolding to coherently reflect and expand on the application of these everyday realms of social and cultural meaning and action.

Anchored in in‐depth qualitative research, the meta‐themes look to the broader settings and concerns of people's lives beyond values or beliefs about IPs per se and the terms on which experts represent them. They do not represent a suggestion of flight from active and better resourced action regarding IPs. Nor do they implicitly position lay people's knowledge and experience as a panacea for IP governance and management. Rather, they expand the basis on which governance and management might proceed. By recognizing the realities and value of lay experience and practice, the meta‐themes demonstrate the important role qualitative research can play in moving toward more reflective, sustained, and ultimately more effective IP governance and management. As such the insights from this meta‐ethnography constitute a platform for much‐needed “mutual dialogue” (Courchamp et al., 2017) in IPs governance and management.

## AUTHOR CONTRIBUTIONS

N.G. conceived the study, led the analysis, and wrote the paper. All authors participated in the analysis as described in the methods. J.A. undertook the first steps of the synthesis stage. G.H. undertook the analysis of the outputs from each stage of the meta‐ethnography. L.C., J.A., S.G., L.H., G.H., and S.M. assisted in preparing the manuscript.

## Supporting information

Appendix S1. Definitions
Appendix S2. Search terms
Appendix S3. Primary database selection ‐ Scopus and Web of Science
Appendix S4. Characteristics of the nineteen papers included in the meta‐ethnography
Appendix S5. An example of phase four mapping of reader analysis of each paper by the relevant sub‐themes
Appendix S6. The nine sub‐themes derived in draft form from the readers’ analyses in phase four, and validated and described in more detail in phase five
Appendix S7. Example of Role of the State sub‐theme with readers’ concepts/issues and examples from papers – as used in phase 5
