# Supplementary material for: Scaling up qualitative research to harness the capacity of lay people in invasive plant management
Source: Conserv Biol. 2022 Jul 27;36(6):e13929. doi: 10.1111/cobi.13929 (PMC10087763; doi:10.1111/cobi.13929)
Supplement: Supplementary file 1 — Appendix S1. Definitions Appendix S2. Search terms Appendix S3. Primary database selection ‐ Scopus and Web of Science Appendix S4. Characteristics of the nineteen papers included in the meta‐ethnography Appendix S5. An example of phase four mapping of reader analysis of each paper by the relevant sub‐themes Appendix S6. The nine sub‐themes derived in draft form from the readers’ analyses in phase four, and validated and described in more detail in phase five Appendix S7. Example of Role of the State sub‐theme with readers’ concepts/issues and examples from papers – as used in phase 5 [file COBI-36-0-s001.docx]

**Supporting Appendices: Scaling up qualitative research to harness the capacity of lay people in invasive plant management**

| **Appendix S1. Definitions** | |
| --- | --- |
| **Concept** | **Scope** |
| Environmental governance | An umbrella term that refers to “the set of regulatory processes, mechanisms and organizations through which political actors influence environmental actions and outcomes…It includes the actions of the state and, in addition, encompasses actors such as communities, businesses, and NGOs. Key to different forms of environmental governance are the political economic relationships that institutions embody and how these relationships shape identities, actions, and outcomes” (Lemos & Agrawal 2006) |
| Environmental management | If governance represents the “steering” mechanisms and principles, environmental management includes the strategies and plans, arrangements, actions and outcomes, and resourcing that arise from environmental governance (Lockwood et al. 2010). |
| Culture | “the production, circulation, and consumption of meanings that become embodied and embedded in social practice” (Storey 2014) |
| Everyday life | This refers to the daily, lived realities and activities of individuals, households, and communities, and the self-evident (to them) subjective experience of everyday life.  “A realm associated with ordinary, routine and repetitive aspects of social life [such as practices, shared meanings, knowledge, rationalities and reasonings, interactions, connections, relationships, routines], that are pervasive and yet frequently overlooked and taken-for-granted” (Gregory et al. 2009) |

| **Appendix S2. Search terms** |
| --- |
| Invasive plants AND Managers AND Social Science  Invasive plants AND Public AND Social Science  Invasive plants AND Public AND Social Science AND Review  Invasive plants AND Managers AND Social Science AND Review  Invasive plants AND Managers AND Review  Invasive plants AND Public AND Review  Invasive plants AND Social Science AND Review  Invasive plants AND Perceptions  Invasive Plants AND Managers AND Attitudes  Invasive Plants AND Public  Invasive Plants AND Public AND Attitudes  Invasive Plants AND Living with  Invasive Plants AND Management AND Public  Invasive Plants AND Farmers  Invasive Plants AND Rural  Invasive Plants AND Interviews  Invasive Plants AND landowner  Invasive Plants AND landholder  Invasive Plants AND private  Invasive Plants AND Attitudes  Invasive Plant Management AND Qualitative  Qualitative AND Invasive plants  Interviews AND Invasive Plants  Invasive Species AND Qualitative (Limit to social Science Journals)  Invasive Species AND Social Science (Limit to social Science Journals)  Invasive Species AND Public (Limit to social Science Journals)  Invasive Species AND Interviews (Limit to social Science & Arts and Humanities Journals)  Invasive Species AND Landowner (Limit to social Science Journals)  Invasive Animals AND Public  Invasive Animals AND Qualitative  Invasive Species AND Rural (Limit to Social Science Journals)  Invasive Plants AND Human Geography  Invasives AND landowner  Invasives AND Rural  Invasives AND Qualitative  Invasives AND Social Science  Invasive plant management AND Social Science  Invasions AND Plants AND Management  Invasions AND Plants AND Landowner |

| **Appendix S3. Primary database selection - Scopus and Web of Science** | |
| --- | --- |
| **Coverage or Issue** | **Description (in summary)** |
| Overall coverage | Web of Science and Scopus are similar but Web of Science is more selective (Singh et al. 2021); Scopus includes most journals indexed by Web of Science (Mongeon & Paul-Hus 2016); in searches both may introduce bias to natural sciences, engineering, and biomedical research and should not be used alone (Mongeon & Paul-Hus 2016); field specific databases should complement their use (Mongeon & Paul-Hus 2016). |
| Science coverage | Web of Science has stronger science coverage (Mongeon & Paul-Hus 2016). |
| Social science coverage | Scopus has stronger social science coverage (Mongeon & Paul-Hus 2016). |
| Access | Web of Science results are influenced by details of institutional subscription that may not be apparent to users (Gusenbauer & Haddaway 2020). |
| Ensuring coverage of ecological and related disciplines in searches | Scopus indexes 820 journals in biological and ecological science disciplines; Scopus indexes relevant journals in these disciplines such as Conservation Biology, Biological Conservation, and Biological Invasions. |

| Appendix S4. Characteristics of the nineteen papers included in the meta-ethnography | | | | |  |  |
| --- | --- | --- | --- | --- | --- | --- |
|  | | | | |  |  |
| Paper No. | **Source paper** | **Country** | **Qualitative methods of data collection** | **Lay participants** | **Land cover/use** | **Species mentioned** |
| 1 | Head, L. & Muir, P. Nativeness, Invasiveness, and Nation in Australian Plants. The Geographical Review 94, 199-217 (2004). | Australia | 259 semi-structured interviews | Suburban backyarders | Suburban gardens | Pittosporum undulatum, Lantana carnara Cinnarnornum camphora |
| 2 | Zagorski, T., Kirkpatrick, J. B. & Stratford, E. Gardens and the Bush: Gardeners’ Attitudes, Garden Types and Invasives. Australian Geographical Studies 42, 207-220, (2004). | Australia | 25 structured interviews | Home gardeners | Suburban gardens | Chrysanthemoides monilifera, Ilex aquifolium, Erigeron karvinskianus, Rubus fruticosus, Prunus domestica, Cotoneaster spp. |
| 3 | Trigger, D. & Mulcock, J. Native vs exotic: cultural discourse about flora, fauna and belonging in Australia. WIT Transactions on Ecology and the Environment 84, 1301–1310 (2005). | Australia | 30 extended interviews, 38 short interviews | Students, people with interest in native plants/animals, people attending a native plant sale, local residents | Urban | Not specified |
| 4 | Evans, J. M., Wilkie, A. C. & Burkhardt, J. Adaptive Management of Nonnative Species: Moving Beyond the “Either-Or” Through Experimental Pluralism. Journal of Agricultural and Environmental Ethics 21, 521-539, (2008). | USA | 24 in-depth interviews | Residents/local water quality subcommittee members | Coastal waterways, multiple terrestrial land uses | Eichhornia crassipes, Hydrilla verticillata |
| 5 | Klepeis, P., Gill, N. & Chisholm, L. Emerging amenity landscapes: Invasive weeds and land subdivision in rural Australia. Land Use Policy 26, 380-392, (2009). | Australia | 3 extended site-visits, 36 semi-structured interviews | Landholders, government officials, weed officers, residents, Landcare members. | Ranching and rural residential | Nassella trichotoma |
| 6 | Brenner, J. C. Pasture Conversion, Private Ranchers, and the Invasive Exotic Buffelgrass (Pennisetum ciliare) in Mexico's Sonoran Desert. Annals of the Association of American Geographers 101, 84-106, (2011). | Mexico | 61 interviews | Large private ranchers | Ranching | Pennisetum ciliare |
| 7 | Selge, S., Fischer, A. & van der Wal, R. Public and professional views on invasive non-native species - A qualitative social scientific investigation. Biological Conservation 144, 3089-3097, (2011). | Scotland | 3 interviews, 9 focus groups (79 participants) | Members of the public (urban dwellers and rural residents), conservation volunteers | Not applicable | Not specified |
| 8 | Fischer, A. P. & Charnley, S. Private Forest Owners and Invasive Plants: Risk Perception and Management. Invasive Plant Science and Management 5, 375-389, (2012). | USA | 60 interviews and property walks | Nonindustrial private forest owners (NIPFs) | Private forest lands | Cirsium arvense, Centaurea spp., Bromus tectorum, Centaurea  Solstitialis, Taeniatherum caputmedusae, Linaria  Dalmatica, Carduus nutans, Euphorbia esula, Salvia aethiopis |
| 9 | Davis, D. & Carter, J. Finding common ground in weed management: Peri-urban farming, environmental and lifestyle values and practices in southeast Queensland, Australia. Geographical Journal 180, 342-352, (2014). | Australia | 10 interviews | Ex-urban landholders, natural resource management volunteers | Rural residential and peri-urban farming | Not specified |
| 10 | Qvenild, M., Setten, G. & Skår, M. Politicising plants: Dwelling and invasive alien species in domestic gardens in Norway. Norsk Geografisk Tidsskrift - Norwegian Journal of Geography 68, 22-33, (2014). | Norway | 22 interviews and garden visits | Domestic gardeners | Domestic gardens in urban, rural, and forested areas | Lupinus polyphyllus, Lysimachia punctata, Stallaria media, Fallopia japonica, Impatiens glandulifera, Rosa rugosa |
| 11 | Doody, B. J., Perkins, H. C., Sullivan, J. J., Meurk, C. D. & Stewart, G. H. Performing weeds: Gardening, plant agencies and urban plant conservation. Geoforum 56, 124-136, (2014). | New Zealand | 16 interviews and garden tours, 90 garden visits | Residents of an 'urban forest' suburb | Suburban gardens | Digitalis purpurea, Convolvulus arvensis, Acer pseudoplatanus, several common garden weeds |
| 12 | Jevon, T. & Shackleton, C. Integrating Local Knowledge and Forest Surveys to Assess Lantana camara Impacts on Indigenous Species Recruitment in Mazeppa Bay, South Africa. Human Ecology 43, 247-254, (2015). | South Africa | 30 interviews | Community elders (65years+) | Forest, farming | Lantana camara |
| 13 | Ernwein, M. & Fall, J. J. Communicating invasion: understanding social anxieties around mobile species. Geografiska Annaler: Series B, Human Geography 97, 155-167, (2015). | Switzerland | 13 interviews, 4 focus groups | A non-expert population, ecological experts | Not applicable | No specific focus or listing but Heracleum mantegazzianum is mentioned |
| 14 | Graham, S. & Rogers, S. How Local Landholder Groups Collectively Manage Weeds in South-Eastern Australia. Environmental Management 60, 396-408, (2017). | Australia | 20 semi-structured interviews | Landcare members, government officials | Farming, rural residential | Nassella trichotoma |
| 15 | Shackleton, S. E. & Shackleton, R. T. Local knowledge regarding ecosystem services and disservices from invasive alien plants in the arid Kalahari, South Africa. Journal of Arid Environments 159, 22-33, (2018). | South Africa | 180 semi-structured interviews | Rural farming householders on streets with declared invasive plants |  | Morus alba, Opuntia  ficus-indica, Prosopis spp., Casuarina cunninghamiana, Eucalyptus camaldulensis, Jacaranda mimosifolia, Leucaena leucocephala, Melia azedarach, Schinus mole, Tecoma stans |
| 16 | Ma, Z., Clarke, M. & Church, S. P. Insights into individual and cooperative invasive plant management on family forestlands. Land Use Policy 75, 682-693, (2018). | USA | 23 semi-structured interviews | Family forest owners, forestry professionals | Private forest lands | Rosa multiflora, Lonicera maacki, Alliaria petiolate, Elaeagnus  umbellata |
| 17 | Cooke, B. & Lane, R. Plant–Human Commoning: Navigating Enclosure, Neoliberal Conservation, and Plant Mobility in Exurban Landscapes. Annals of the American Association of Geographers 4452, 1-17, (2018). | Australia | 39 interviews with 45 participants, property walks | Exurban landholders | Rural residential, hobby farming, private land conservation | No specific focus or listing but Rubus fruticosus agg., and “pines” are listed |
| 18 | Bach, T. M., Kull, C. A. & Rangan, H. From killing lists to healthy country: Aboriginal approaches to weed control in the Kimberley, Western Australia. Journal of Environmental Management 229, 182-192, (2019). | Australia | 94 interviews, participant observation over three years | Aboriginal traditional owners (elders) | Multiple tenures and uses in urban and non-urban remote areas | Leucaena leucocephala, Parkinsonia aculeata, Passiflora foetida, Macroptilium atropurpureum, Azadirachta indica, Cenchrus ciliaris, Cenchrus biflorus, Tribulus terrestris, Calctropis procera, Jatropha gossypifolia, Ziziphus mauritiana, Hibiscus sabdariffa, Colocasia esculenta, Andropogon gayanus |
| 19 | Shrestha, B. B. et al. Community perception and prioritization of invasive alien plants in Chitwan-Annapurna Landscape, Nepal. Journal of environmental management 229, 38, (2019). | Nepal | 32 focus groups with 218 participants | Executives and general members of Community Forest Users' Groups, local farmers | Community forests, conservation areas and national parks, farming | Ageratum houstonianum, Oxalis latifolia, Pistia stratiotes, Argemone Mexicana, Galinsoga quadriradiata, Parthenium hysterophorus, Ipomoea carnea ssp. Fistulosa, Lantana camara, Ageratina Adenophora, Spermacoce alata, Ageratum houstonianum, Chromolaena odorata, Bidens Pilosa, Parthenium hysterophorus, Lantana camara, Mimosa pudica, Mikania micrantha, Chromolaena odorata, Ageratina Adenophora, Lantana camara, Mikania micrantha |
| Totals | **Year range: 2004-2019** | **9 countries** | **1,019 interviews with 1,025 participants; 45 focus groups with approximately* 318 participants** | **Participants include landowners in rural, urban and peri/ex-urban areas; private ranchers and forest owners; community elders; farmers; traditional owners; members of voluntary land and forest stewardship groups; university students; and general residents and members of the public** |  |  |
| *Paper 13 provided a range of participants per focus group rather than an exact overall number. We have averaged this information. | | | | |  |  |

| **Appendix S5. An example of phase four mapping of reader analysis of each paper by the relevant sub-themes** | | | | | | | |
| --- | --- | --- | --- | --- | --- | --- | --- |
| **PAPER 8. Fischer and Charnley 2012. "Private Forest Owners and Invasive Plants: Risk Perception and Management." Invasive Plant Science and Management 5: 375-389.** | | | | | | | |
| **Role of the state** | **Agency/**  **performance** | **Social relations** | **Plant-human relationships** | **Blame and responsibility** | **Tensions/**  **differences/gaps** | **lay knowledges/values/**  **concerns** | **Other** |
| Governance currently mitigates against collective management, better governance needed to foster it | Agency of government organisations is as much recognised in what they do as what they don't do, paper suggests they are not cognisant of this. Is the State ignoring its responsibilities? | Both farmers and non-farmers inhabit areas for similar reasons including lifestyle, amenity, nature, and income, authors posit this as post-productivist and argue landcare doesn't successfully bridge the divide between landholders. | Paper is against separatist views of nature v culture, and agricultural v environmental concerns | There was a lot of shifting blame to neighbours’ lack of management and the difficulty of the politics of this problem. People want someone they perceive as not having an agenda 'other than to protect the landscape and the landscapes future' to take responsibility. | The sense of what it means to look after weeds is variable, sense of what one sees as harmful is going to vary | Time priorities put IS management as low | Authors of paper concerned with 'production and protection' of peri-urban landscape |
| participants recognise government neglect of public land being similar to absentee landowners and other people with little time, non farmers don’t see government as representing them |  | weeds don’t respect property boundaries - collective action is needed through coordinated responses amongst government, community, & landholders. Landholders acknowledged this needs to be done at the landscape scale. |  | How one sees weeds will depend on one’s stewardship orientation |  | conflicts of living with weeds amongst different landholders |  |
| landholders see a lack of political will to prioritise or fund weed management esp. on public and absentee lands that get abandoned |  | separatist gaze towards peri-urban landholders are unhelpful, instead focus on collaborative/unified responses and points of intersection (which authors suggest are more apparent than previously acknowledged/utilised) |  |  |  |  |  |
| the government needs to see itself as a landholder with its own intersecting values |  | Differences between landowner types can be overstated, they are disposed toward collective management more than it is being utilised. |  |  |  |  |  |

| **Appendix S6. The nine sub-themes derived in draft form from the readers’ analyses in phase four, and validated and described in more detail in phase five** | | | |
| --- | --- | --- | --- |
| **Sub-theme** | **Description (in summary)** | **Issues** | **Papers the sub-theme relates to** |
| Lay knowledges, values, and concerns | Lay perspectives and relationship especially relative to official knowledge | Local or indigenous environmental knowledge relative to “official” or expert knowledge; heterogeneous, multi-faceted and sometimes in tension with itself; how people make sense of their worlds and invasive plants; not conventionally “rational” but have their own rationalities. | Eighteen papers: 1, 2, 3, 4, 5, 6, 7, 8 ,9 ,10, 11, 12, 13, 14, 15, 16, 18, ,19 |
| Plant-human relationships | Significance of the relationships between plants and humans for invasive plant management. | How invasive plants are assimilated into lives and livelihoods; wide variety of relationships with invasive plants; relationships vary across scales. | Fourteen papers: Nos. 1, 2, 3, 4, 5, 8, 9, 10, 11, 12, 14, 16, 17, 18 |
| Agency and performance | Decentering human agency relative to plant agency; decentering state agency relative to agency of lay people | Plant agency key to formation of plant-human relationships; the world is multi-agentic; hierarchies of human-plant and expert-lay challenged; lay management a “performance” or emergent outcome of plant/human agency | Fourteen papers: Nos. 1, 4, 6, 7, 8, 9, 10, 11, 13, 14, 15, 16, 17, 18 |
| Tension and differences | Tensions and differences between and within groups | Conflict or differences between state and lay perspectives; conflict and differences among lay groups; role of different interests; trade-offs and tensions in tolerance and action regarding invasive plants. | Thirteen papers : Nos 2, 3, 4, 5, 6, 7, 8, 9, 10, 12, 14, 16, 18. |
| Control | Who controls invasive plants and how and why | Are plants controllable? What level of control is possible? What are the goals? Futility; Can endless invasive plant labour be meaningful and how? What comes after letting go of control? | Ten papers: Nos. 5, 6, 7, 8, 10, 11, 14, 16, 17, 18 |
| Social relations | Relations among groups and relevance for invasive plant management | Invasive plant management as a public good; role of collective and landscape scale management; Identity, independence, and interest in sociability. | Ten papers: Nos. 5, 6, 8, 9, 11, 12, 14, 15, 16, 18 |
| Role of the state | Including role relative to lay people, and how people view the state and its various officials, policies, and actions and absences | Differences, tensions, trust, roles, priorities, and power and authority; agencies and local government don’t manage their own land; lack of resources shows true government position on invasive plants. | Nine papers: Nos. 6, 8, 9, 14, 15, 16, 17, 18, 19 |
| Responsibility and blame | Questions around who is responsible for invasive plants and who is meeting/not meeting responsibilities | Responsibility to consider needs of others and meet invasive plant management obligations; blaming others; blame invokes moralities of responsibility, laziness vs industriousness, and care/lack of care for others; if blame is about distinctions, care is about connections; moral obligation of humans to address invasive plants and care for the earth. | Seven papers: Nos, 6, 7, 8, 9, 14, 16, 17 |
| Nomenclature issues | Differences around language and terminology for invasive plants | Consequences of disjuncture between expert/government discourses/terminology and lay discourses/experience; binary thinking and language (e.g. native/non-native) versus variable and contextual lay values and experience of invasive plants. | Seven papers: Nos. 1, 4, 7, 10, 13, 17, 18 |

| **Appendix S7. Example of Role of the State sub-theme with readers’ concepts/issues and examples from papers – as used in phase 5** | | |
| --- | --- | --- |
| **Readers’ concepts/issues that relate to role of the state** | **Examples from papers** | **Paper** |
| **agency** | Structure/agency balance important - so far too much focus on role of the state. | 6 |
| **agency** | Need to acknowledge ranchers as primary agents of pasture conversion, not the state. | 6 |
| **bridging the gap between state and lay people** | All the landcare groups acknowledged that part of their success arose from “boundary spanners” who helped them to negotiate bureaucratic processes. They also identiﬁed a role for governments in engaging with landholders who did not contribute to group goals; sanctioning was seen as outside the scope of group activities. (12) | 14 |
| **collective management** | Governance currently mitigates against collective management, better governance needed to foster it | 9 |
| **collective management** | what is the role of the state in helping collective management capacity? | 14 |
| **differences amongst lay groups** | Different ranchers have different values regarding government support | 6 |
| **differences amongst lay groups** | Some think state should be responsible for public land and owners for private; others see invasive plants as requiring state action across tenures. | 16 |
| **distrust of government** | Ranchers can be wary of participating in official programs due to oversight and feeling financially obligated to others. | 6 |
| **distrust of government** | Non farmers do not see government as representing them and addressing their interests in land. | 9 |
| **funding** | Managing weeds requires integrative approach and funding and coordination at multiple jurisdictions. For this area, stronger regulation and compliance needed. Findings suggest that "professional management and stronger regulation are not viable in the short term". | 5 |
| **funding** | Landholders see a lack of political will to prioritise or fund weed management esp. on public and absentee lands that get abandoned | 9 |
| **funding** | Funding from government is important, not simply 'capacity building' in other ways. | 14 |
| **human-plant relations** | Rethinking state metrics through context and socio-natural relationships (human-plant-space) | 18 |
| **importance of independence** | Ranchers take pride in their independence and are cynical about government assistance. | 6 |
| **importance of independence** | Voluntary incentives over legislative ones favoured but unclear on role of state in assisting private landowners | 16 |
| **importance of independence** | Don't want legislation, want to be independently responsible | 16 |
| **landscape scale** | Weeds do not respect property boundaries and therefore collective action is needed through coordinated responses amongst government, community, and landholders, this was acknowledged by landholders as needing to be done at the landscape scale. | 9 |
| **landscape scale** | State responsible for cross-boundary management and cooperation? | 16 |
| **landscape scale** | Role of the state in framing conservation and landscapes | 17 |
| **national policy** | Interested in national level quantifying of economic costs of IS | 19 |
| **national policy** | Authors want to influence national policy | 19 |
| **responsibility** | Most gardeners had 'touching faith' in education and community awareness but none mentioned regulation as an option. Authors argue awareness without regulation will not solve the problem. | 2 |
| **responsibility** | Different garden types represented different values – “Native” gardeners = local government should do more to influence/regulate gardening practice. “Woodland” gardeners = government should do more, nurseries also a problem. “Gardenesque” gardeners and “shrub” gardeners didn't mention government | 2 |
| **responsibility** | Agency of government organisations is as much recognised in what they do as what they don't do, paper suggests they are not cognisant of this. Is the State ignoring its responsibilities? | 9 |
| **responsibility** | Considers the role of the state in introducing species | 15 |
| **state as just another agent in a multi-agentic world** | Participants recognise government neglect of public land being similar to absentee landowners and other people with little time | 9 |
| **state as just another agent in a multi-agentic world** | the government needs to see itself as a landholder with its own intersecting values | 9 |
| **state interests** | Government sees many benefits of buffelgrass | 6 |
| **state interests** | State distribute forage with weed seeds in it | 19 |
| **tension between state views and other views of weed management** | Differences found between state agency views of problem species and lay views | 8 |
| **tension between state views and other views of weed management** | State enforced weed management v. Aboriginal understanding of 'healthy country' | 18 |
| **tension between state views and other views of weed management** | Hierarchy of State challenged - State should listen and implement based on Aboriginal understandings/values, not the other way around. Scott's Seeing like a State relevant - people, nature, places are made 'legible' to state logic, whereas it should be other way. | 18 |
| **tension between state views and other views of weed management** | Strategies for control need rethinking to move away from futility and frustration of weed management implemented by the state | 18 |

Gregory, D., R. Johnston, G. Pratt, M. Watts, and S. Whatmore 2009. The Dictionary of Human Geography. Wiley-Blackwell, Hoboken.

Lemos, M. C., and A. Agrawal. 2006. Environmental Governance. Annual Review of Environment and Resources **31**:297-325.

Lockwood, M., J. Davidson, A. Curtis, E. Stratford, and R. Griffith. 2010. Governance Principles for Natural Resource Management. Society & Natural Resources **23**:986 - 1001.

Mongeon, P., and A. Paul-Hus. 2016. The journal coverage of Web of Science and Scopus: a comparative analysis. Scientometrics **106**:213-228.

Singh, V. K., P. Singh, M. Karmakar, J. Leta, and P. Mayr. 2021. The journal coverage of Web of Science, Scopus and Dimensions: A comparative analysis. Scientometrics **126**:5113-5142.

Storey, J. 2014. From popular culture to everyday life. Routledge, London ;.
